# Dispensing Antibiotics without Prescription at Community Pharmacies and Accredited Drug Dispensing Outlets in Tanzania: A Cross-Sectional Study

**DOI:** 10.3390/antibiotics10081025

**Published:** 2021-08-23

**Authors:** Pendo M. Ndaki, Martha F. Mushi, Joseph R. Mwanga, Eveline T. Konje, Nyanda E. Ntinginya, Blandina T. Mmbaga, Katherine Keenan, Wilber Sabiiti, Mike Kesby, Fernando Benitez-Paez, Alison Sandeman, Matthew T. G. Holden, Stephen E. Mshana, HATUA Consortium

**Affiliations:** 1 Department of Biostatistics, Epidemiology and Behavioral Sciences, School of Public Health, Catholic University of Health and Allied Sciences, Mwanza P.O. Box 1464, Tanzania; pendo.ndaki@bugando.ac.tz (P.M.N.); ekonje28@bugando.ac.tz (E.T.K.); 2 Department of Microbiology and Immunology, Weill Bugando School of Medicine, Catholic University of Health and Allied Sciences, Mwanza P.O. Box 1464, Tanzania; marthamushi@bugando.ac.tz (M.F.M.); mshana72@Bugando.ac.tz (S.E.M.); 3 Mbeya Centre, National Medical Research Institute, Mbeya P.O. Box 2410, Tanzania; nelias@nimr-mmrc.org; 4 Kilimanjaro Christian Medical Centre, Kilimanjaro Clinical Research Institute, Kilimanjaro Christian Medical University College, Moshi P.O. Box 2236, Tanzania; b.mmbaga@kcri.ac.tz; 5 Geography and Sustainable Development Department, University of St. Andrews, St. Andrews KY16 9AL, UK; katherine.keenan@st-andrews.ac.uk (K.K.); mgk@st-andrews.ac.uk (M.K.); fernando.benitez@st-andrews.ac.uk (F.B.-P.); 6 Division of Infection and Global Health, School of Medicine, University of St. Andrews, St. Andrews KY16 9AL, UK; ws31@st-andrews.ac.uk (W.S.); as7@st-andrews.ac.uk (A.S.); mtgh@st-andrews.ac.uk (M.T.G.H.)

**Keywords:** antibiotic, antibiotic resistance, dispensing practice, prescription

## Abstract

Worldwide, antimicrobial resistance is increasing rapidly and is associated with misuse of antimicrobials. The HATUA study (a broader 3-country study) investigated the antibiotic dispensing practices of pharmaceutical providers to clients, particularly the propensity to dispense without prescription. A cross-sectional study using a ‘mystery client’ method was conducted in 1148 community pharmacies and accredited drugs dispensing outlets (ADDO) in Mwanza (*n* = 612), Mbeya (*n* = 304) and Kilimanjaro (*n* = 232) in Tanzania. Mystery clients asked directly for amoxicillin, had no prescription to present, did not discuss symptoms unless asked [when asked reported UTI-like symptoms] and attempted to buy a half course. Dispensing of amoxicillin without prescription was common [88.2, 95%CI 86.3–89.9%], across all three regions. Furthermore, the majority of outlets sold a half course of amoxicillin without prescription: Mwanza (98%), Mbeya (99%) and Kilimanjaro (98%). Generally, most providers in all three regions dispensed amoxicillin on demand, without asking the client any questions, with significant variations among regions [*p*-value = 0.003]. In Mbeya and Kilimanjaro, providers in ADDOs were more likely to do this than those in pharmacies but no difference was observed in Mwanza. While the Tanzanian government has laws, regulations and guidelines that prohibit antibiotic dispensing without prescription, our study suggests non-compliance by drug providers. Enforcement, surveillance, and the provision of continuing education on dispensing practices is recommended, particularly for ADDO providers.

## 1. Introduction

Since their discovery in 1928, antibiotics (ABs) have revolutionized treatment of illnesses caused by microorganisms and contributed significantly to decrease in mortality worldwide. However, the global expansion of ABs use has been paralleled by a similar increase in antimicrobial resistance (AMR) [1]. A systematic evaluation of antibiotic consumption by the World Health Organization (WHO) across 76 countries over a 15-year period (2000–2015) revealed overall use per capita increased by 26% in the first- and second-line therapies and 91% in those used only with specific indication due to high resistance [2]. In Tanzania, between 2017 and 2019, antimicrobial consumption for all antimicrobials was reported to be 80.8 ± 39.35 defined daily doses (DDDs) per 1000 inhabitants, with amoxicillin having a DDD/1000 of 16.75, ranking second after doxycycline as the most commonly consumed antimicrobial. The total consumption was greater than in many high-income countries in Europe, Japan and China, however there was no data to compare from Sub-Saharan African countries [3,4]. In addition, in 2020, data from six referral hospitals using a point prevalence survey (PPS) observed that approximately 62.3% of inpatients were prescribed antibiotics [5]. Globally, in both healthcare and community settings, resistance is increasing in bacteria that cause many common diseases and the WHO now lists antimicrobial resistance as one of the top ten threats to global health [6].

A 2015 WHO study of 12 low and middle-income countries (LMICs) showed that 93% of patients obtain antibiotics from medical stores and pharmacies leaving only a minority who obtain antibiotics directly from health professionals [7]. Furthermore, many LMIC patients view drug sellers in small drug shops and community pharmacies, as a more accessible first point of call for health care seeking for common disease treatment and, crucially, antibiotic provision [8]. Health or care-seeking behavior has been defined as any action undertaken by individuals who perceive themselves to have a health problem or to be ill for the purpose of finding an appropriate remedy [9].

Despite prohibitive regulation, there is considerable evidence indicating that ABs are commonly dispensed without prescription and/or appropriate advice in many LMICs [10]. Over-the-counter medication with ABs can delay hospital admission, mask the diagnosis of infectious diseases, and facilitate the development and spread of resistant pathogens. The willingness of providers to make ABs available without prescription is itself one of the factors that drive over-the-counter medication [11].

In Tanzania, several reports have been published that advocate more appropriate, or so-called ’rational’ use of antibiotics [12]. Studies conducted on the development of antimicrobial stewardship programs in Tanzania have stated the need to increase investments in the medicine regulatory authority to achieve this [13]. The Tanzania Medicine and Medical Devices Authority (TMDA) which mandates the licensing, monitoring, and regulating of drugs in Tanzania, categorizes community pharmacies as either type 1 pharmacies or type 2 pharmacies/accredited drugs dispensing outlets (ADDO). Type 1 pharmacies are supposed to operate under the supervision of a registered pharmacist, with certain conditions (e.g., air-conditioning, refrigeration of drugs and a dispensing window) and can sell all three categories of retail medicines: prescription only (POM) which includes antibiotics, pharmacy only (PO), and general sale list (GSL). ADDOs do not require a registered pharmacist and are supervised by any person who has attended a five weeks’ ADDO training course [14]. Another key difference between pharmacies and ADDOs is that the latter are more restricted in terms of the drugs they can sell. ADDOs are permitted to sell only medicines on the GSL (which includes for example common painkillers, cold and flu remedies), and can dispense some antibiotics with prescription, for instance, amoxicillin capsule/suspension, benzyl-penicillin powder for injections, chloramphenicol eye drops, Trimethoprim/Sulfamethoxazole suspension, doxycycline capsules/tablets, Phenoxymethylpenicillin suspension/tablets and procaine penicillin fortified [15].

The ADDO program was a donor-initiated project launched in 2003 to improve pharmaceutical access in underserved communities and aimed to regulate local retail drug shops (previously known as maduka ya dawa baridi), which were often located in rural, and peri-urban settings [16]. As of 2019, there were 1504 Type 1 registered pharmacies and 14,045 ADDOs in Tanzania [17] which are now known in Kiswahili as ‘maduka ya dawa muhimu’ (literally, essential medicines shops). Reviews suggest that ADDOs are central to health-seeking behavior, being widely accepted as convenient, reliable, and preferable sources of medication by local communities and help to improve antibiotic access [18]. However, there are some reports of inappropriate over-dispensing of antimicrobials in ADDOs [18]. As self-medication with antimicrobials seems relatively common in Tanzania, as in many LMIC settings, the role of ADDOs in fostering antibiotic overuse of ABs should be further investigated [19].

There have been relatively few studies on community pharmacy and ADDO antibiotic provision in Tanzania, and these are limited geographically and dated [20,21,22,23]. A mystery client study in the urban area of Moshi (Kilimanjaro, northeast Tanzania) found 92% of community pharmacies provide antibiotics without prescription [24], and client exit interviews in urban Moshi found three-quarters of antibiotic purchases were made without prescription [22]. Studies evaluating ADDO practices specifically have revealed similarly high rates of inappropriate antibiotic provision. One study of mystery client visits in ADDOs across Tanzania found one-third of ADDOs sold antibiotics for cold-like symptoms, and 85% sold antibiotics on request without prescription [18]. In 2011, a mystery client study of 145 providers in the Morogoro region reported 79% of ADDOs sold drugs without prescription [25]. Further evidence is required to assess whether these patterns can be generalized to the rest of the country, or if they vary systematically by pharmacy type, and between rural and urban areas. Such variation has been observed in other LMIC settings including other parts of East Africa [26] and Vietnam [27]. In this paper, we report the results of a mystery client study with drug sellers in three different regions of Tanzania, which aimed to investigate variations in antibiotic dispensing practices by area and type of pharmacy, and the quality of advice provided by the antibiotic seller.

## 2. Materials and Method

### 2.1. Study Design

This study is nested within a wider 3-country interdisciplinary study on the drivers of AMR in East Africa (Holistic Approach to Unravel Antibacterial Resistance in East Africa—HATUA), and full details can be read elsewhere [28]. This cross-sectional community-based quantitative survey uses mystery client scenarios, sometimes called mystery clients or mystery shoppers to assess dispensing practices. Mystery client studies are a commonly used tool in LMIC settings to observe drug provider behavior first-hand and minimize observation bias [2]. In our study this involved trained field workers posing as clients who visited pharmacies and ADDOs and enacted a scenario that tested a specific behavior (in this case AB dispensing practices) [29].

### 2.2. Study Areas and Sampling

**Figure 1:**
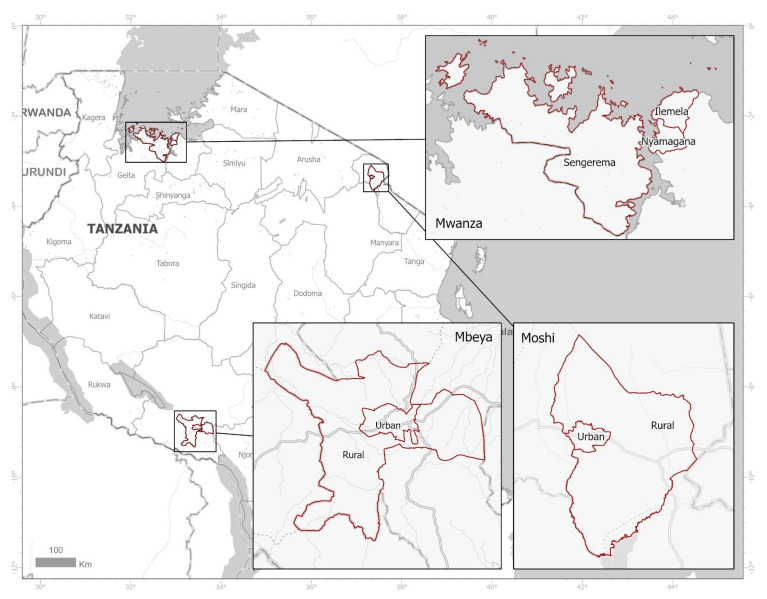
Location of our three study areas within Tanzania.

This cross-sectional study was carried out in the Mwanza (Nyamagana, Ilemela and Sengerema), Mbeya (Mbeya urban and Mbeya rural districts) and Kilimanjaro (Moshi urban and Moshi rural districts) regions of Tanzania between April and July 2019. The regions and respective districts were purposely selected based on the geographical location to cover northeastern Tanzania, northwestern Tanzania and South-western highlands of Tanzania [28]. Figure 1 shows the geographical location of regions. The main economic activities of Mwanza are subsistence farming, fishing and livestock keeping, while in Mbeya and Kilimanjaro they are mainly subsistence farming. Nyamagana and Ilemela District form the city of Mwanza (the second largest in Tanzania). Sengerema, Mbeya District Council and Moshi District Council represent the rural population while Ilemela, Nyamagana, Mbeya City Council and Moshi Municipal Council represent the urban areas.

**Figure 2:**
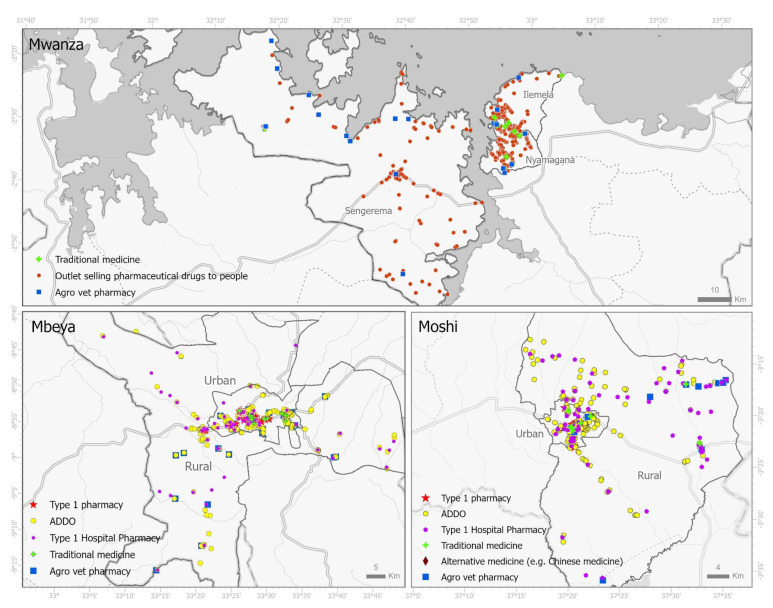
Geographical distribution of ADDOs, pharmacies and other drug providers mapped in the three study areas. ADDOs and pharmacies were surveyed in the mystery client study.

To create a comprehensive sampling frame for the study, we undertook a mapping exercise within our study areas (Mwanza 25–29 March, Mbeya 5–9 May and Kilimanjaro 20–24 May). Teams of trained fieldworkers systematically traversed roads and pedestrian areas, recording the location, type of outlet and observable regulatory compliance (e.g., display of certificates) of all formal and informal medicine outlets, using GPS-enabled tablets and a report form developed in the research software package Epicollect5 [30]. Figure 2 shows the locations of retail pharmaceutical outlets.

The global positioning system (GPS) coordinates generated by the mapping provided a list of pharmacies and ADDOs that formed the sample frame for the mystery client study. To avoid selection bias and to maximize the sample size, the study aimed to survey 100% of the previously mapped type 1 community pharmacies and ADDOs. Given the focus on human antibiotic provision, we excluded agro vets and alternative/traditional medicine shops, and we also excluded 9 shops because they were closed on the day of the survey. The survey was piloted in Misungwi district in Mwanza and all entries were reviewed to confirm fieldworker compliance with the protocol, and practical challenges were addressed before further data collection commenced in April 2019 (Mwanza) and May 2019 (Mbeya and Kilimanjaro) using assigned lists from the sample frame to avoid repetition.

### 2.3. Data Collection

The mystery clients (MCs) comprised 25 research assistants (10 females and 15 males) unknown by the drug sellers, the majority of whom were undergraduate pharmacy students and the remainder other health students. MCs received training on the study objectives, protocol and sampling strategy, ethical issues, and the mystery client scenario (see the Supplementary Materials S1). Visiting each seller once, MCs requested amoxicillin and, unless questioned by the seller, did not describe symptoms (of UTI) or state that they did not have a prescription. If the seller refused to sell or advised a non-antibiotic remedy, the MCs took that advice and bought what was recommended and left. If the seller was willing to dispense amoxicillin in these circumstances or recommended another pharmaceutical drug instead or as well, the MCs then requested that they be allowed to buy only a “couple of days’ worth” of the drug(s) to ‘see if their friend’s advice was good/it worked’. If the seller insisted they buy a full course, the MCs did so.

The full course was defined according to the Tanzania Standard Treatment Guidelines [15] which state an adult minimum course of amoxicillin of 500 mg 8-hourly for 5 days which translates to 30 capsules of amoxicillin. In addition, a full course should be whatever a doctor had prescribed, and sellers should ask for a prescription before selling the antibiotic. After the encounter, fieldworkers moved away from the premises and recorded what transpired, inputting data directly into Epicollect5 on their tablets [30].

### 2.4. Data Analysis

The results were analyzed using descriptive univariate and bivariate statistics. The practice of dispensing across regions was reported as a proportion with a 95% confidence interval.

The main binary outcome of this study was selling amoxicillin without prescription. We also created a summary score variable that measured, in the context of the given scenario, the quality of the interaction between client and seller and the degree to which practices among sellers were in line with AB stewardship programs. We used the following list of binary variables:

(1) either advised buying a full course, or would only sell a full course, (2) asked for a prescription, (3) asked if the MC was taking any other medications, (4) suggested that the MC did not need antibiotics, (5) recommended that the MC see a doctor and/or get a prescription, (6) gave clear instructions (oral or written) that the MC should buy a full course (even if they eventually sold a half course) and (7) gave clear instructions (oral or written) that the MC should normally finish a full course (even if they eventually sold a half course). The scores ranged from 0 to 7. We used bivariate contingency tables to assess differentials in these outcomes across the three regions and districts, and by type of pharmacy. The strength of bivariate associations was tested using Chi-square tests or Fisher’s exact tests (depending on cell sample size). The data collected were downloaded from Epicollect5 and checked for consistency before being transferred to Stata version 13 (Stata Corp., College Station, TX, USA).

### 2.5. Ethical Considerations

The study received ethical approval from the University of St. Andrews, UK (No. MD14548, 10/09/19); National Institute for Medical Research, Tanzania (No. 2831, updated 26/07/19), Mbeya Medical Research and Ethics Committee (No. SZEC-2439/R.A/V.1/30), Kilimanjaro Christian Medical College, Tanzania (No. 2293, updated 14/08/19) and CUHAS/BMC research ethics and review committee (No. CREC/266/2018, updated on 02/2019). While sellers did not consent, the scenario did not require sellers to do or say anything outside of what they would otherwise be doing as part of their usual practice. Furthermore, the responses gathered at particular sites and from particular individuals, are not identifiable in the data presented.

## 3. Results

### 3.1. Community Pharmacies and ADDOs in Mwanza, Mbeya and Kilimanjaro 

A total of 612, 304 and 232 community pharmacies and ADDOs were mapped in Mwanza, Mbeya, and Kilimanjaro, respectively (Table 1). The population density per drug outlet was higher in rural areas than urban areas: the highest densities were found in Kilimanjaro rural (4094) followed by Mwanza rural (3489) (Table 1). In rural areas, ADDOs outnumbered pharmacies almost 100-fold (except Mbeya where they were 10 to 1), and in Urban areas they did so by more than three-fold (except Mwanza).

### 3.2. Stocking, Dispensing and Price of Amoxicillin in Mwanza, Mbeya and Kilimanjaro

Almost all community pharmacies and ADDOs (1084, 94.0%) stocked amoxicillin during the study period. This was observed across all three regions: Mwanza (563, 91.0%), Mbeya (297, 97.0%), and Kilimanjaro (224, 96.0%). Of these, the majority dispensed amoxicillin ‘on demand’ without posing any question to the client (see Table 2). Of even greater significance, the vast majority in all regions responded positively, without further question or comment, to the MC’s request that they dispense ‘only a few days’ worth’.

The price of one capsule of amoxicillin (250 mg) in all regions was 100Tsh (0.043 USD). The analysis of 708 sellers with full details revealed that the number of capsules dispensed in response to MC’s request for ‘a few days’ (half course) varied considerably (1–20 capsules), with a median of 10 capsules (one blister pack) (IQR: 5–10). The number of capsules dispensed for a ‘full course’ was consistent (30 capsules) and conformed exactly to Tanzanian regulation for a minimum adult course for amoxicillin. Full course without prescription was dispensed in only 6 drug outlets.

Only a small minority of those prepared to sell a half course told the MC that the consumption of a full course was advisable. Very few denied the request and insisted on selling a full course (the highest was 8.0% Mwanza). These results were not affected by researcher bias; MCs only made one request for a half course and did not attempt to persuade the seller.

Furthermore, across the three sites, whilst no MC held a prescription, an overwhelming majority of sellers dispensed amoxicillin as requested by the MC [88.2%, 95%CI 86.3–89.9%], although there was some significant variation by region [Mwanza, 85.3%, 95%CI 82.3–87.9%, Mbeya, 90.3%, 95%CI 86.6–93.3% and Kilimanjaro, 93.1%, 95%CI 89.0–95.7%] and this difference (between Mwanza compared to Mbeya and Kilimanjaro) was statistically significant (chi2 = 11.851 and *p*-value = 0.003).

### 3.3. Other Antibiotics Dispensed without Prescription

The MC scenario protocol required the MCs to ask for amoxicillin (with no prescription) and only if questioned, to describe UTI symptoms, an ailment for which amoxicillin, if prescribed, would have been an appropriate treatment. Nevertheless, in a small minority of cases, sellers dispensed another antibiotic that had not been requested, instead of amoxicillin. Ampicloxacillin and ciprofloxacin were the most frequently sold alternatives (Table 3).

### 3.4. Differences between ADDOs and Pharmacies Regarding Dispenisng Practices

**Figure 3:**
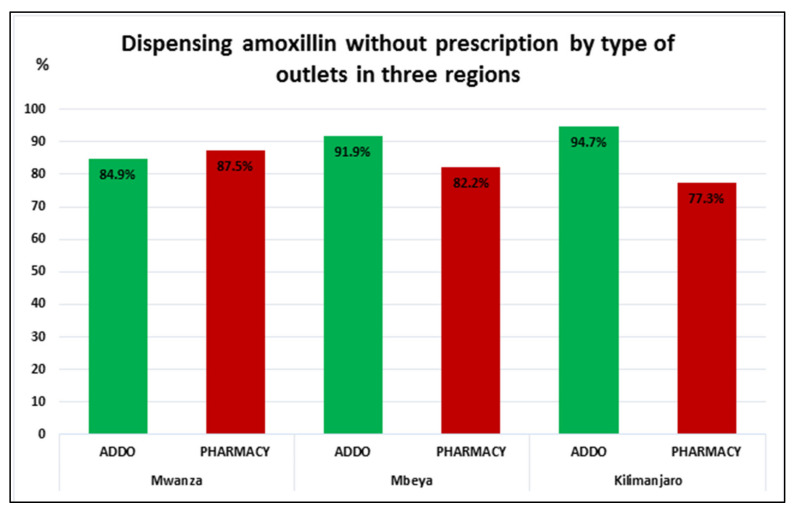
Proportion of sellers dispensing without prescription, according to types of outlets across three regions.

Cognizant of the significant difference in the absolute numbers of ADDOs vs. community pharmacies (see Figure 3), we disaggregated dispensing amoxicillin without prescription by seller type. No significant difference was observed in terms of dispensing between ADDOs and community pharmacies in Mwanza region (84.9% vs. 87.5%, chi2 = 0.357 and *p* = value 0.550), and while dispensing without prescription was higher in ADDOs in Mbeya (96.9% vs. 82.2%, chi2 = 4.154 and *p* = 0.042) and Kilimanjaro (94.7% vs. 77.3, chi2 = 9.486 and *p* = 0.002), over two-thirds of pharmacies dispensed without prescription in all regions.

With regard to the quality of interaction recorded in Table 2 (questions asked and advice given), the difference between ADDOs and pharmacies was as follows: in Mbeya and Kilimanjaro, 17.8% and 22.7%, respectively, of pharmacies addressed at least one of the seven question or advice items before selling amoxicillin compared to only 8.1% and 5.2% of the sellers from ADDOs. However, no statistical difference was observed between different types of sellers in Mwanza (community pharmacies 12.5%, ADDOs 15.04% chi2=0.357 and *p* = value 0.550) (Figure 3).

**Figure 4:**
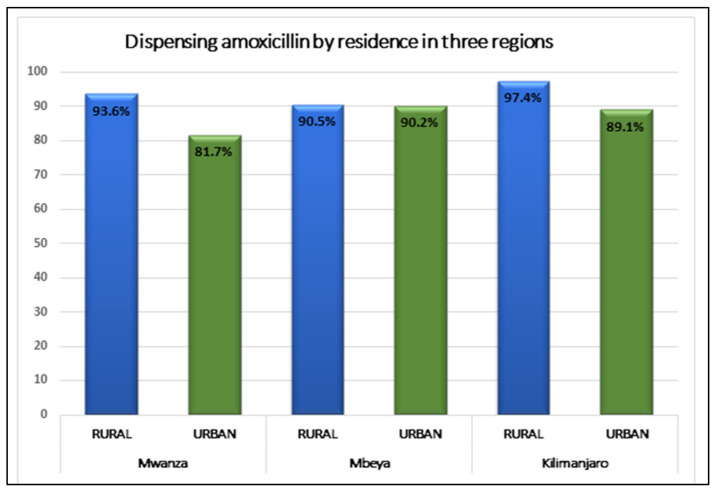
Location of outlets dispensing amoxicillin without prescription in three regions.

Figure 4 shows differences in dispensing amoxicillin according to urban/rural areas of the three regions. Whilst in Kilimanjaro and Mwanza, the likelihood of selling antibiotics without prescription was over 10% higher among rural outlets compared with those located in urban areas, the differences were not statistically significant. The likelihood of selling ABs was between 82–9% in all areas (Figure 4).

## 4. Discussion

As part of a wider three-country investigation of the drivers of AMR in East Africa, this study aimed to explore the role played by private drug shops in the antibiotic provision landscape in three sites in Tanzania (Mwanza, Mbeya and Kilimanjaro regions). The Tanzania Food, Drugs and Cosmetics (Scheduling of Medicines) Regulations [13] prohibit the sale of antibiotics without prescription. We investigated how different kinds of drug sellers, in different contexts, would respond to a request for a common antibiotic (amoxicillin) from a client who did not have a prescription and who did not initially describe their symptoms (indicative of a UTI).

Our findings indicate that more than 90% of all community pharmacies and ADDO surveyed across the three regions dispensed antibiotics without prescription. This is higher than both the figures reported for Africa (62%) and North America (74%) in a recent 38 study systematic review [31] and the findings of a previous study conducted in Morogoro, Tanzania (85%) [25]. Study design might explain some of the difference in the findings. Our MC scenario was based on a condition for which a doctor might reasonably write an antibiotic prescription (e.g., probable UTI infection), whilst other studies sometimes describe conditions that would not. Additionally, it should be noted that our MCs were both men and women, and recommended treatment for UTI can differ between males and females. However, in both cases, it is worth remembering that few sellers in our study asked the relevant questions that would allow them to identify the MC’s condition (less than 1% asked to see a prescription and less than 10% asked about symptoms). Therefore, our study seems to corroborate the findings of a 2018 urban-only study in Kilimanjaro region that reported 92.3% of surveyed community pharmacies dispensed antibiotics without prescription [24] and extends these findings to cover rural areas as well.

Our scenario tested not only the outcome of a request for amoxicillin without prescription but also the quality of the interaction between sellers and clients at the point of sale. Across the study, interactions were recorded as almost universally poor with only 11.75% of all sellers either asking questions or offering any advice to the client. Quality of interaction scores was differentiated by site, with Mwanza region having 90 (14.7%) of sellers with an interaction score of 1–7, whilst Mbeya had 29 (9.5%) and Kilimanjaro only 16 (6.9%) (*p*-value 0.003). In all regions, less than 1.5% of sellers asked either for a prescription or checked if clients were taking other medications. While instructions to buy or finish a full course were common suggestions among the minority that asked any questions or gave any advice (6.4%), this did not prevent most from dispensing a half course, nevertheless. The better (but still poor) quality of interaction scores in Mwanza might relate to the presence of a medical university offering a diploma in pharmaceutical sciences and a bachelor’s in pharmacy and the likelihood that students work part-time in some community pharmacies, but only further research could confirm this. The quality of interaction findings for this study, whilst specific to a particular context, are comparable with similar studies in other least developed and middle-income countries, for example, an Ethiopian study in which 11.8% of 422 sellers asked at least one question [32] and a Pakistani study that recorded 11% of 353 sellers asked about medication history [33]. However, all these regional case studies compare poorly with a study from Shenyang, northern China (upper middle-income country) in which 82.3% asked at least one question [34]. The lack of sustained monitoring of pharmacy conduct by the authorized bodies is among the likely reasons for the poor level of seller-client interactions observed in this study.

Importantly, our study not only investigated whether sellers would dispense antibiotics without prescription (something others have tested before), it also tested whether sellers would dispense a quantity of drugs below the regulated minimum-, or ‘full-’ course. This was an important question since sale of a partial dose may further compound the risk of AMR development. We found that 93.03% of sellers granted the client’s request for ‘only a few days’ worth.’ Of these, while a smaller number sold as ‘a half course,’ a quantity close to the ‘full course,’ the majority sold half or less of that quantity. Only six sellers insisted that they buy a full course.

Comparing our data on both urban and rural areas, sellers in rural areas were more likely to dispense amoxicillin without prescription than those in urban areas. In Mwanza, of 425 outlets located in urban areas, 84% sold amoxicillin without prescription compared to 92% of 187 outlets located in rural areas, *p* = 0.014. Significant difference was also observed in Kilimanjaro (urban *n* = 214, 93.3% vs. rural *n* = 90, 100%, *p* = 0.003). No significant difference was observed in Mbeya (urban *n* = 425 and rural *n* = 187). This is similar to that reported in a recent Vietnamese study (88% urban and 91% rural) [35] where, whilst a very different context, more sellers in rural than urban areas were also dispensing antibiotics without prescription. Some of the rural-urban difference in Tanzania might be explained by the potentially greater difficulty of enforcing regulations in rural areas, or that there is less health care and laboratory infrastructure and fewer qualified personnel to appropriately implement and enforce the regulations [16]. However, on these grounds, it is surprising that the difference between rural and urban areas is not greater than is observed (see Figure 4).

Meanwhile, sale of antibiotics without prescription was higher in ADDO shops than in community pharmacies. An explanation for this disparity might be sought in the likely differential in the level of pharmacy education of the persons operating the shops. In Tanzania law, a Part I pharmacy must be supervised by the registered pharmacist whilst a part II pharmacy can be supervised by anyone who has completed the five weeks’ training on accredited drugs dispensing outlets (ADDO) [12]. However, these significant differences in qualification do not seem to translate into radically different dispensing practices; the lowest rate for pharmacies selling without prescription was 77% (Kilimanjaro) in Mwanza and Mbeya it was over 80%. Therefore, something other than qualifications, or lack thereof, must be driving the common practice of dispensing without a prescription.

One possible driver of the observed prescription practice in this study may be the relative abundance of drug sellers and the likely impact this has on market forces. Such a distribution was one of the criticisms leveled at the “Duka La Dawa Baridi” (DLDB) structure of small drug shops that the ADDO regulation was created to replace [16]. More than this, whereas regulations require a spatial separation of 500 m between pharmacies and 300 m between ADDOs in small towns and 200 m in rural areas [14] our study recorded instances where these separations were not respected. A forthcoming paper will explore these geographic drivers in more detail.

Recently, Tanzania was the first African country to be recognized by the WHO as having achieved a well-functioning regulatory system for medical products (achieved through Tanzania Food and Drugs Authority (TFDA) now known as Tanzania Medicines and Medical Devices Authority (TMDA) [14]. If fully implemented, these regulations should ensure patient safety and curtail the development of AMR. However, while the infrastructure of regulation may exist, the evidence from our study demonstrates that under objective test conditions, the majority of sellers were willing to breach regulations and sell prescription antibiotics to a client who did not possess a prescription, and furthermore, most were prepared to sell a half course, with very few giving the advice that completion of a full course was recommended. Clearly, continuous training about antibiotic stewardship and antibiotic resistance, stricter enforcement of regulation and/or an increased acquiescence to rules is required if Tanzania’s acclaimed system is to contribute effectively to the fight against AMR. However, further research is also needed on what motivates clients/patients to demand, and sellers to supply, antibiotics over the counter without prescription. Forthcoming work from the HATUA consortium that analyses qualitative interviews and focus groups with patients, community members, doctors, and drug sellers, as well as impact of the geographic proximity of sellers, hopes to illuminate these questions further.

## 5. Limitations

First, our MCs were both male and female, and yet UTI treatment for men and women is often different. However, the scenario used in the current study required the mystery client to request amoxicillin in the first instance and only report UTI symptoms if questioned. Second the scenario used in the current study required the mystery client to request amoxicillin rather than presenting symptoms and asking for a seller’s recommendation. A scenario based on presenting symptoms and asking for advice on treatment may have resulted in more, or less dispensing of antibiotics and or a greater variety of antibiotics dispensed. A HATUA Phase 2 MC survey will test such a scenario. Third, each seller was visited only once, however, the consistency of response across multiple sellers offsets the lack of multiple visits to anyone seller. Fourth, the short delay between the MCs interaction in a drug shop, and their completion of the Epicollect log at a short distance from the shop is open to the possibility of recall bias. Fifth, within the limits of this paper it has not been possible to fully address the data on the geographic proximity of sellers. This will be addressed in forthcoming papers.

## 6. Conclusions

This project used a mystery client study (MCS) to explore the dispensing practices of private drug providers (both community pharmacies and ADDOs), in response to a scenario in which a client, with no prescription, and not initially describing symptoms, asked for the antibiotic amoxicillin, and subsequently for a partial course. It did so via a near 100% sample of sellers in both the rural and urban areas of three regions of Tanzania (Mwanza, Mbeya and Kilimanjaro). The study’s contribution is to offer, from multiple sites and on a scale not yet seen in Tanzania, objective data on antibiotic dispensing practices, and also to offer insights on the quality of seller-client interaction at the point of sale; both of which can inform policy around antibiotic stewardship in this and other majority world contexts.

The study findings reveal that unprescribed antibiotics are commonly dispensed and sold in community pharmacies and ADDOs across the country. It demonstrates that while such dispensing is more common among ADDOs than community pharmacies, and in rural areas (where ADDOs predominate) than in urban areas, it is nevertheless the most common practice in all sectors, contexts, and regions. Furthermore, the study reveals that (in the context of the tested scenario), the quality of the interaction between sellers and clients, in terms of clinically relevant questions asked and antibiotic stewardship relevant advice given is almost universally poor.

Further research is needed to better understand the motivations of providers and consumers, and how these vary by location and regulatory context. This will require more subtle MCS scenarios, for example, that test seller responses to the description of symptoms likely to benefit from a course of antibiotics, rather than a straightforward demand of a given drug, to test whether dispensing without a prescription remains common and whether the quality of questioning and advice improves.

The detailed knowledge emerging from this (and the broader HATUA) study is vital in planning interventions to address the problems of inappropriate demand and provision of antibiotics and ultimately, antibiotic resistance.

Our recommendations based on this study are that immediate action needs to be taken by the Ministry of Health Community Development, Gender, Elderly and Children in creating awareness and understanding on antibiotic resistance through effective information, education, and communication among different audiences in the community including school settings, village assemblies, health facilities and political gatherings. This will help in promoting behavior change for both antibiotic sellers and consumers.

## Figures and Tables

**Table 1:** Number of drug outlets and density per head of population by region and district.

Study Region/District	Population (Average House Size)	Number of Drug Shops			Outlet Density per Population (Region)	Outlet Density per Population (District)
		ADDO	PHARMACY	Total		
**Mwanza**					2205.3	
Urban	706453 (4.8)	324	110	434		1600.3
Rural	663,034 (6.0)	184	3	187		3489.7
**Mbeya**					2271.7	
Urban	385,279 (4.2)	142	31	173		1888.6
Rural	305,319 (4.1)	120	11	131		2150.1
**Kilimanjaro**					2593.7	
Urban	184,292 (4.0)	97	23	120		1288.8
Rural	466,737 (4.2)	110	2	112		4094.2

**Table 2:** Sellers’ dispensing practice and quality of interaction across the three regions.

Seller Response when Directly Asked to Sell a Half Course of Amoxicillin	Mwanza (*n* = 612)	Mbeya (*n* = 304)	Kilimanjaro (*n* = 232)	TOTAL (*n* = 1148)	Chi-Square and *p* Value
Did not sell amoxicillin ^a^	47 (7.7)	3 (1.0)	7 (3.0)	57 (4.9)	Pearson chi2(2) = 21.267, Pr = 0.000
Sold half course without questions	509 (83.2)	276 (90.8)	216 (93.1)	1001 (87.2)	Pearson chi2(2) = 19.652 Pr =0.000
Sold half course but advised full course	45 (7.4)	22 (7.2)	6 (2.6)	73 (6.4)	Pearson chi2, 6.9546 Pr = 0.031
Would only sell a full course	11 (1.8)	3 (1.0)	3 (1.3)	17 (1.5)	Fisher’s exact = 0.554
**Quality of interaction by item**					
Asked about prescription	9 (1.5)	2 (0.7)	3 (1.3)	14 (1.2)	Fisher’s exact = 0.631
Asked to describe symptoms	54 (8.8)	16 (5.3)	13 (5.6)	83 (7.22)	Pearson chi2(2) = 5.851 Pr = 0.054
Asked about taking other medications	10 (1.6)	4 (1.3)	3 (1.3)	17 (1.5)	Fisher’s exact = 1.000
Suggested no need of antibiotics	5 (0.8)	1 (0.3)	3 (1.3)	9 (0.8)	Fisher’s exact = 0.492
Suggested to see a doctor	11 (1.8)	0	9 (3.9)	20 (1.7)	Fisher’s exact = 0.001
Provided instruction to buy a full course	49 (8.0)	19 (6.3)	6 (2.6)	74 (6.4)	Pearson chi2(2) = 9.0799 Pr = 0.011
Provided instruction to finish a full course	47 (7.7)	20 (6.9)	6 (2.6)	73 (6.4)	Pearson chi2(2) = 7.721 Pr = 0.021
**Quality of interaction additive score**					
0 - no question asked or advice given	522 (85.3)	275 (90.5)	216 (93.1)	1013 (88.2)	Pearson chi2 = 11.851Pr = 0.003
1 question or piece of advice	35 (5.7)	6 (1.9)	4 (1.7)	45 (3.9)	
2 questions or pieces of advice	28 (4.6)	14 (4.6)	9 (3.9)	51 (4.4)	
3 questions or pieces of advice	18 (2.9)	8 (2.6)	0	26 (2.3)	
4 questions or pieces of advice	6 (0.9)	1 (0.3)	0	7 (0.6)	
5 questions or pieces of advice	2 (0.3)	0	0	2 (0.2)	
6 questions or pieces of advice	1 (0.2)	0	0	1 (0.1)	
7 questions or pieces of advice	0	0	3 (1.3)	3 (0.3)	
Total with a score between 1–7	90 (14.7)	29 (9.5)	16 (6.9)	135 (11.7)	

^a^ ‘Did not sell’ could be ‘refused to sell’, or ‘did not stock’, or ‘sold alternative drug instead of amoxicillin’.

**Table 3:** List of alternative drugs sold and number of sellers who sold them.

Mwanza (*n =* 26)	Mbeya (*n* = 13)	Kilimanjaro (*n* = 1)
Ampicloxacillin (9)	Ampicloxacillin (1)	Trimethoprim/sulfamethoxazole (1)
Ampicillin (3)	Ampicloxacillin and Phenoxymethylpenicillin (1)	
Metronidazole (2)	Azithromycin and Metronidazole (1)	
Azithromycin (2)	Cephalexin (1)	
Ciprofloxacin (5)	Ciprofloxacin (4)	
Doxycycline (1)	Doxycycline and Metronidazole (1)	
Trimethoprim/sulfamethoxazole (3)	Nitrofurantoin and Azithromycin (1)	
Doxycycline and Azithromycin (1)	PenV (1)	
	Trimethoprim/sulfamethoxazole (1)	
	Trimethoprim/sulfamethoxazole (1)	

## Data Availability

Not applicable.

## Supplementary Materials

The following are available online at https://www.mdpi.com/article/10.3390/antibiotics10081025/s1. S1: Mystery Clients Scenario.
