# Supplementary material for: Dispensing Antibiotics without Prescription at Community Pharmacies and Accredited Drug Dispensing Outlets in Tanzania: A Cross-Sectional Study
Source: Antibiotics (Basel). 2021 Aug 23;10(8):1025. doi: 10.3390/antibiotics10081025 (PMC8389015; doi:10.3390/antibiotics10081025)
Supplement: Supplementary file 1 [file antibiotics-10-01025-s001.zip › antibiotics-1308212-supplementary.pdf]

The Mystery Client Scenario.

Scenario protocol: The ‘patient’ requests antibiotics

1. Ask directly for amoxicillin (or other basic antibiotic)
2. If asked for a prescription, say you do not have one (but do not mention this unless asked) .
3. If asked for an explanation for the requested antibiotic say that you have been told by a friend that this drug is good for treating your problem.
4. Do not give your symptoms unless asked. Only if asked, report a burning sensation when urinating.
5. If you are asked whether you are taking any other medication, say ‘no’.
6. If the drug is available and the seller indicates they will sell you the drug, say that – in the first instance you only wish to buy a couple of days’ worth to see if your friend’s advice was good.
7. If the seller says that drug is not available – or not available without proscription - then ask for the seller’s advice (again do not discuss your symptoms unless the seller invites you to)
8. Take the seller’s advice: (a) If they suggest you do not need antibiotics or recommend a non-antibiotic remedy, accept this advice, buy what is recommended and leave: (b) If they recommend a different drug or an additional drug, accept this advice, but again attempt to buy only a couple of days-worth say ‘Ill see if that helps me improve before I decide to get anymore’. (c) If they recommend that you buy a full course do so.
